# Microglial Mincle receptor in the PVN contributes to sympathetic hyperactivity in acute myocardial infarction rat

**DOI:** 10.1111/jcmm.13890

**Published:** 2018-10-24

**Authors:** Yu Wang, Jie Yin, Cailing Wang, Hesheng Hu, Xiaolu Li, Mei Xue, Ju Liu, Wenjuan Cheng, Ye Wang, Yan Li, Yugen Shi, Jiayu Tan, Xinran Li, Fuhong Liu, Qiang Liu, Suhua Yan

**Affiliations:** ^1^ School of Medicine Shandong University Jinan China; ^2^ Department of Cardiology Qianfoshan Hospital of Shandong Province Jinan China; ^3^ Department of Endocrinology Qianfoshan Hospital of Shandong Province Jinan China; ^4^ Medical Research Center Qianfoshan Hospital of Shandong Province Jinan China

**Keywords:** IL‐1β, microglia, Mincle, myocardial infarction, NLRP3, PVN, sympathetic hyperactivity

## Abstract

Malignant ventricular arrhythmias (VAs) following myocardial infarction (MI) is a lethal complication resulting from sympathetic nerve hyperactivity. Numerous evidence have shown that inflammation within the paraventricular nucleus (PVN) participates in sympathetic hyperactivity. Our aim was to explore the role of Macrophage‐inducible C‐type lectin (Mincle) within the PVN in augmenting sympathetic activity following MI,and whether NOD‐like receptor family pyrin domain‐containing 3 (NLRP3) inflammasome/IL‐1β axis is involved in this activity. MI was induced by coronary artery ligation. Mincle expression localized in microglia within the PVN was markedly increased at 24 hours post‐MI together with sympathetic hyperactivity, as indicated by measurement of the renal sympathetic nerve activity (RSNA) and norepinephrine (NE) concentration. Mincle‐specific siRNA was administrated locally to the PVN, which consequently decreased microglial activation and sympathetic nerve activity. The MI rats exhibited a higher arrhythmia score after programmed electric stimulation than that treated with Mincle siRNA, suggesting that the inhibition of Mincle attenuated foetal ventricular arrhythmias post‐MI. The underlying mechanism of Mincle in sympathetic hyperactivity was investigated in lipopolysaccharide (LPS)‐primed naïve rats. Recombinant Sin3A‐associated protein 130kD (rSAP130), an endogenous ligand for Mincle, induced high levels of NLRP3 and mature IL‐1β protein. PVN‐targeted injection of NLRP3 siRNA or IL‐1β antagonist gevokizumab attenuated sympathetic hyperactivity. Together, the data indicated that the knockdown of Mincle in microglia within the PVN prevents VAs by attenuating sympathetic hyperactivity and ventricular susceptibility, in part by inhibiting its downstream NLRP3/IL‐1β axis following MI. Therapeutic interventions targeting Mincle signalling pathway could constitute a novel approach for preventing infarction injury.

## 1 INTRODUCTION

Ventricular arrhythmias (VAs) following acute myocardial infarction (AMI) are common complications which remain a major cause of mortality, and primarily result from sympathetic nerve hyperactivity.^1^ The paraventricular nucleus (PVN) is a nucleus controlling cardiovascular regulation and is located in the hypothalamus of the central nervous system(CNS).^2^ Recent studies have demonstrated that inflammation within the PVN is related to regulation of the cardiovascular sympathetic tone,^3^, ^4^, ^5^ however, the precise mechanism remains largely unknown.

Innate immunity makes a great sense to protect host against pathogens’ invasion,^6^, ^7^ as well as in the pathological process of several diseases including MI. Various pattern recognition receptors (PRRs) are activated, indicating the initiation of innate immunity.^6^ Macrophage‐inducible C‐type lectin (Mincle), also identified as C‐type lectin domain family 4 (CLEC4E), is a kind of transmembrane germline‐encoded PRR^8^ and is expressed on surface of various immune cells comprising monocytes/macrophages, neutrophils and others.^9^ It could be activated via recognizing a kind of endogenous ligand named Sin3A‐associated protein 130 kDa (SAP130) which belongs to subunit of the histone deacetylase.^10^ The downstream protein spleen tyrosine kinase (Syk) is phosphorylated after activation of Mincle receptor, followed by the various routes to synthesize pro‐inflammatory cytokines like IL‐1β and chemokines,^10^ resulting in the infiltration of macrophages and phagocytizing dying cells.^11^ Previous studies have demonstrated that Mincle is recognized as a launching point in various noninfectious inflammatory diseases such as traumatic brain injury and subarachnoid haemorrhage (SAH).^10^, ^12^ However, the role of Mincle in MI has not yet been explored.

Several studies have reported that the activation of microglia and an increase in pro‐inflammatory cytokines are observed in the PVN post‐MI.^13^, ^14^ Among all of the pro‐inflammatory cytokines, IL‐1β is recognized as a key trigger of inflammation. It is released following the activation of microglia in CNS and is involved in sympathetic hyperactivity.^15^ The nucleotide‐binding and oligomerization domain‐like receptor family pyrin domain‐containing 3 (NLRP3) inflammasome containing of apoptosis speck‐like protein containing a caspase‐recruitment domain (ASC), NLRP3 and pro‐caspase‐1 is reported to participate in cleaving the IL‐1β precursor to form the mature and secreted structure.^16^, ^17^ Simultaneously one previous study reported that Mincle receptor plays an important role in NLRP3 inflammasome formation and IL‐1β synthesis by activated myeloid cells.^18^

Mincle receptor is primarily expressed in microglia known as the monocyte‐macrophage cell lineage of the CNS.^19^, ^20^ A direct role for Mincle and microglia derived from the PVN in sympathetic nerve excitation and the induction of ventricular arrhythmias post‐MI has not been explored. Our aim is to investigate whether Mincle within the PVN could participate in enhancing sympathetic nerve excitability via NLRP3/IL‐1β pathway post‐MI.

## 2 MATERIALS AND METHODS

### 2.1 Animals

Male Sprague‐Dawley rats (50‐60 days of age, weighting approximately 280 g; Vitalriver Company; Beijing, China) were raised in a thermostatic (22°C) room with constant relative humidity (50%). They received humane care with a 12 hours light/12 hours dark cycle (light from 6:00 to 18:00). All the experimental protocols and procedures used in this study were performed in accordance with the Animal Care Committee of Shandong University Affiliated with Qianfoshan Hospital (Protocol number: S 030). The experiments were implemented after the rats adapted to their new environment for approximately 7 days.

### 2.2 Experimental design

The entire experiments were divided into three separate protocols.

#### 2.2.1 Protocol 1

Seventy‐nine rats survived after MI surgery were divided into seven groups (control, MI after 6, 12, 24 hours, 3, 5, and 7 days, n = 11 each group). The temporal expression of SAP130, Mincle, NLRP3 and IL‐1β were measured by Western blot.

#### 2.2.2 Protocol 2

A total of 187 rats were randomly placed into four groups: (1) sham + scrambled siRNA (Thermo Fisher, Waltham, MA, USA, 250 pmol/50 nL sterile saline), n = 40; (2) sham + Mincle siRNA (Thermo Fisher, 250 pmol/50 nL), n = 42; (3) MI+ scrambled siRNA, n = 50; (4) MI+ Mincle siRNA, n = 55. To promote the gene silencing efficiency, three different sequences targeting Mincle siRNA were selected: (a) 5′CACCUUAUCCUGGCUAUCAAGUCUA3′; (b) 5′GCUCACCUGGUGGUUAUCAACACAU3′; and (c) 5′CCUGUUUCUUCAGUAUGCCUUGGAU3′. The rats received PVN microinjection 24 hours prior to MI surgery.

#### 2.2.3 Protocol 3

112 rats were randomly divided into six groups: (a) naïve, n = 14; (b) scramble siRNA, n = 16; (c) LPS (Sigma‐Aldrich, St. Louis, MO, USA, 12.5 μg of LPS dissolved in 50 nL of saline), n = 17; (d) LPS+rSAP130 (Abnova, Taiwan, China, 25 ng in 50 nL of saline), n = 21; (e) LPS+rSAP130 + NLRP3 siRNA (Thermo Fisher, 250 pmol/50 nL), n = 22; (f) LPS+rSAP130 + gevokizumab (XOMA 052; XOMA Corporation, Emeryville, CA, USA, 50 nL of saline solution containing 10 μg of gevokizumab), n = 22. The doses of reagents were referred according to previous studies.^12^, ^21^, ^22^, ^23^, ^24^ Animals in naïve group did not receive any procedure except anesthesia until RSNA measurement. The rats were stimulated with LPS via PVN microinjection, and 3 hours later recombinant SAP130 (rSAP130) was administrated. To promote the gene silencing efficiency, two different NLRP3 siRNA sequences were selected: (a) 5′GAUCCUAUUUGAAGAGUGU3′; (b) 5′GAUCAACCUCUCUACCAGA3′. The NLRP3 siRNA was injected into the PVN 24 hours prior to LPS stimulation, and pharmacological IL‐1β inhibitor gevokizumab was used immediately after rSAP130 administration. On the time duration, animals which did not receive targeted reagents’ injection were microinjected with same dosage of saline as control to ensure that each rat was microinjected to PVN for three times. Six hours later, the RSNA was recorded, and tissues were collected for ELISA and Western blot analysis.

### 2.3 PVN microinjection

In protocol 2 and 3, the rats were mounted on a stereotaxic apparatus (RWD Life Science, San Diego, CA, USA) after being anaesthetized by 2% sodium phenobarbital sodium (i.p., 40 mg/kg). Two small holes were drilled through the top of the skull approximately 1.8 mm posterior and 0.4 mm lateral to the bregma. A pair of cannulas were implanted on the skull of rat and animals received recovering for 7 days. When microinjected to PVN, the cannulas were connected to two 0.5‐μL microinjectors via a pair of PE‐10 tubes mounted on an infusion pump (RWD Life Science) was inserted through the holes bilaterally into the PVN (7.9 mm from the top of the skull). The prepared reagents were injected bilaterally into the PVN at a rate of 0.1 μL/min and a dose of 50 nL each side. The microinjector was removed slowly after 15 minutes of intermittence.

### 2.4 MI surgery

In protocol 1 and 2, MI models were developed as described previously.^25^ Procedures were performed in a sterile environment and rats underwent a 12 hours of fasting period before and after their surgical procedures. Buprenorphine (0.05 mg/kg) was injected subcutaneously to control the pain after anesthetization, then rats were intubated via tracheotomy and ventilated with a small‐animal ventilator. The heart was exposed via a left thoracotomy at the third and fourth intercostal space and the left coronary artery was ligated 2–3 mm from its origin between the pulmonary artery conus and the left atrium with a 6‐0 polypropylene ligature. In the sham animals, we exposed the heart but did not ligate the artery. Postsurgery, the rats were not returned to their own cages but were placed on a heating pad at 37°C to maintain their body temperature until they woke up. Bupivacaine (1 mg/kg) was administered at the intercostal incision during closure, and penicillin (0.8 million units) was injected intramuscularly to prevent infection. Animals were monitored daily after the surgery. Tissue samples were harvested 24 hours following MI surgery in protocol 2.

### 2.5 HRV measurements

Telemetry was conducted on the rats in protocol 2 after the MI surgery. We placed a radiotelemetry transmitter (TR50B; AD Instruments, Sydney, Australia) body in the abdominal cavity of the rats; the positive lead wire was placed on the xiphoid process, while the negative lead wire was placed between the sternocleidomastoid muscles. The frequency domains were very‐low‐frequency (VLF; 0.05 Hz), low‐frequency (LF; 0.05‐0.75 Hz), and high‐frequency (HF; 0.75‐2.5 Hz) bands. Data from dynamic electrocardiography (ECG) was recorded via the LabChart Pro software (AD Instruments). The ratio of LF to HF was calculated. The ECG was monitored for 24 hours until the RSNA recording.

### 2.6 RSNA recording

The RSNA recording was conducted to monitor the activity of the renal sympathetic nerve after the rats were anaesthetized with 2% phenobarbital sodium (40 mg/kg). The rats were placed in the left lateral position before receiving a left flank incision. The renal sympathetic nerve was separated from the surrounding tissue in the abdominal cavity under an anatomical microscope and hooked to a pair of bipolar silver wire recording electrodes. The electrodes and the nerve were immersed in liquid paraffin to insulate them from the surrounding tissue and to prevent desiccation. An oscilloscope (AD instruments) was used to amplify, filter and monitor the electrical data with a frequency cut‐off from 100 to 3000 Hz. The bipolar platinum electrode was connected to a biological polygraph (AD instruments) to record the RSNA simultaneously. The changes in RSNA were calculated as percentage change from the baseline activity recorded before the experiments. Both the integrated and raw RSNA values are represented in the results; however, only the integrated data were statistically analysed.^26^

### 2.7 In vivo electrophysiological experiments

Programmed electrical stimulation was conducted as previously described. Briefly, the rats were placed in the supine position to receive thoracotomy, and electrodes were implanted on the epicardial surface of the LV.^27^ Eight paced beats were applied at a basic cycle length of 150 milliseconds (S0) to induce VAs, followed by single (S1), double (S2), and triple (S3) additional stimuli at shorter coupling intervals. The stimulation intensity was twice the threshold, and the stimulus length was set to 5 milliseconds. The stimulating protocols were completed within 10 minutes. An arrhythmia scoring system was applied to measure the induced arrhythmic degree. All the protocols were conducted via an animal biological function experiment system (LEAD‐7000; JJET, Chengdu, China).

### 2.8 Tissue collection

An intravenous injection of KCl was administrated to kill the animals following the electrophysiological study, after which they were decapitated, and the whole brain was removed. The brain tissue in each group was collected using different methods. (a) The location of the PVN was determined, and a microinjection of methylene blue was administered in line with the Paxinos and Watson rat atlas^28^ (supplemental data). The PVN was separated from the whole brain tissue and stored at −80°C immediately for further biochemical analysis. (b) The brain tissues were collected and placed in freshly prepared 4% paraformaldehyde for 12 hours at 4°C and transferred to 10% neutral buffered formalin (NBF) until sectioning. (c) The brain tissue was immersed in PBS solution containing 30% sucrose over‐night, embedded in Tissue‐Tek^®^ OCT compound (Sakura Finetek, Tokyo, Japan) and frozen at −80°C before further analysis. The sample size for immunohistochemical studies and immunofluorescence was 2.0 × 1.0 cm^2^. The hearts and blood were additionally harvested. After stored at −80°C for 1 hour, the heart was cut into 2 mm thick slices and stained with 1% triphenyltetrazolium chloride (TTC) for 30 minutes at 37°C. We recognized the infarcted area based on visualization of the pale ventricle wall^29^ and measured it via TTC staining using Image ProPlus 5.0 software (supplemental data). An infarct size between 30% and 50% for each rat was chosen in the study.

### 2.9 Immunohistochemistry

Each brain was cut into 4‐μm‐thick sections for immunohistochemical studies after MI. The slices were incubated with primary antibodies: goat polyclonal antibody against Iba‐1(1:200, Abcam, Cambridge, UK) and anti‐CD34 rabbit polyclonal antibody (1:2500, Abcam) at 4°C overnight, after which DAB substrate (Vector Laboratories, Burlingame, CA, USA) and haematoxylin were used in order for staining.

### 2.10 Immunofluorescence

A freezing microtome (CM3050; Leica, Wetzlar, Germany) was used to cut the brain tissues into 8‐μm‐thick coronal sections. The slices were fixed with 4°C precooling acetone for 10 minutes and incubated with Iba‐1 goat polyclonal antibody (1:150, Abcam) and Mincle rabbit polyclonal antibody (1:200; Bioss corporation, Beijing, China) over night at 4°C. Next, the slices were incubated with secondary antibodies for 2 hours: Alexa 488‐conjugated donkey anti‐rabbit (1:200; Thermo Fisher), Alexa 594‐conjugated donkey anti‐goat (1:150, Thermo Fisher). The cell nuclei were stained with DAPI (Life Technologies, Billerica, MA, USA). Olympus LCX100 Imaging System and ImageJ were used for obtaining images and images analysis, respectively.

### 2.11 Western blot

The PVN tissues were homogenized in RIPA lysis buffer (Beyotime Institute of Biotechnology, Jiangsu, China) containing PMSF (Beyotime Institute of Biotechnology) (ratio of 100:1). A BCA assay kit (Pierce Protein Biology, St. Louis, MO, USA) was used to determine the protein concentration. Approximately 70 μg of total protein for each sample were resolved on 8%‐15% polyacrylamide gels and transferred onto PVDF membranes (Bio‐Rad, Richmond, VA, USA). The membranes were blocked for 1 hour using 5% nonfat dry milk diluted with TBS and then probed with different antibodies overnight at 4°C: anti‐SAP130 rabbit monoclonal (Abcam; 1:2000), anti‐Mincle rabbit polyclonal (Bioss corporation; 1:1000), anti‐NLRP3 rabbit polyclonal (Abcam; 1:500), anti‐IL‐1β rabbit monoclonal (Cell Signaling Technology, Danvers, MA, USA; 1:1000), anti‐Iba‐1 goat polyclonal (Abcam; 1:1000) and anti‐GAPDH rabbit polyclonal (Goodbio Technology, Wuhan, China; 1:1000) antibodies. Following a 2 hours‐incubation with HRP‐conjugated anti‐rabbit or anti‐goat secondary antibodies (GenScript, Piscataway, NJ, USA; 1:5000), and immunodetection was conducted using the ECL (Millipore, Billerica, MA, USA) method. NIH ImageJ software (National Institutes of Health, Bethesda, MD, USA) was used to analyse the grey scale value.

### 2.12 Enzyme‐linked immunosorbent assay (ELISA)

The peripheral blood and myocardium tissues were treated using a double‐antibody sandwich ELISA kit (Cusabio Biotech Co, Wuhan, China) according to the manufacturer's instructions to detect norepinephrine (NE), a marker of sympathetic nerve activity. The NE tissue concentration was reported in pg/mg myocardium tissue. Each treatment was repeated at least three times. The mean values of the concentration of NE were statistically compared among the groups.

### 2.13 Statistics

The data are presented as the means ± standard deviations (SDs). Values for more than two groups were compared using ANOVA followed by Tukey's test using SPSS software, version 19.0 (SPSS Inc., Chicago, IL, USA). Statistical significance was considered as *P *<* *0.05.

## 3 RESULTS

### 3.1 SAP130 and Mincle signalling pathway expression were increased in rats post‐MI

**Figure 1:**
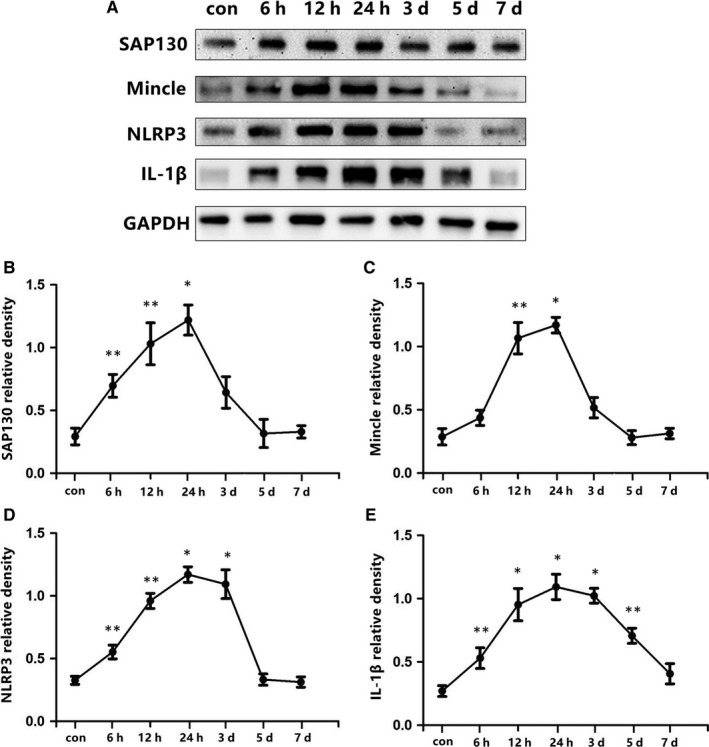
A, Expression profiles of the SAP130 (130 kD), Mincle (25 kD), NLRP3 (120 kD), IL‐1 (17 kD) protein in the PVN by western blot control, 6, 12, 24 h, 3, 5, 7 days post‐MI. SAP130, Mincle, NLRP3 and IL‐1 were quantified relative to the GAPDH (37 kD) levels (B‐E). n = 11‐12 per time‐point. *P < 0.01 and **P < 0.05 vs control group

Western blot was conducted to detect the temporal patterns of SAP130, Mincle, NLRP3, and IL‐1β expression within the PVN post‐MI. We noted that expression of SAP130, the endogenous ligand of Mincle receptor, started to increase as early as 6 hours after MI and lasted till 24 hours, and recovered to the basal level at 3 days post‐MI (Figure 1B). The level of Mincle receptor in the PVN was markedly increased during the 12 hours after ligation of coronary artery and reached a peak at 24 hours (Figure 1C). Increased expression of NLRP3 and IL‐1β were also observed at 6 h and remained at a high level between 3 and 5 days post‐MI (Figure 1D, E).

### 3.2 Locally blockage of Mincle receptor within the PVN inhibited NLRP3/IL‐1β expression and microglial infiltration post‐MI

**Figure 2:**
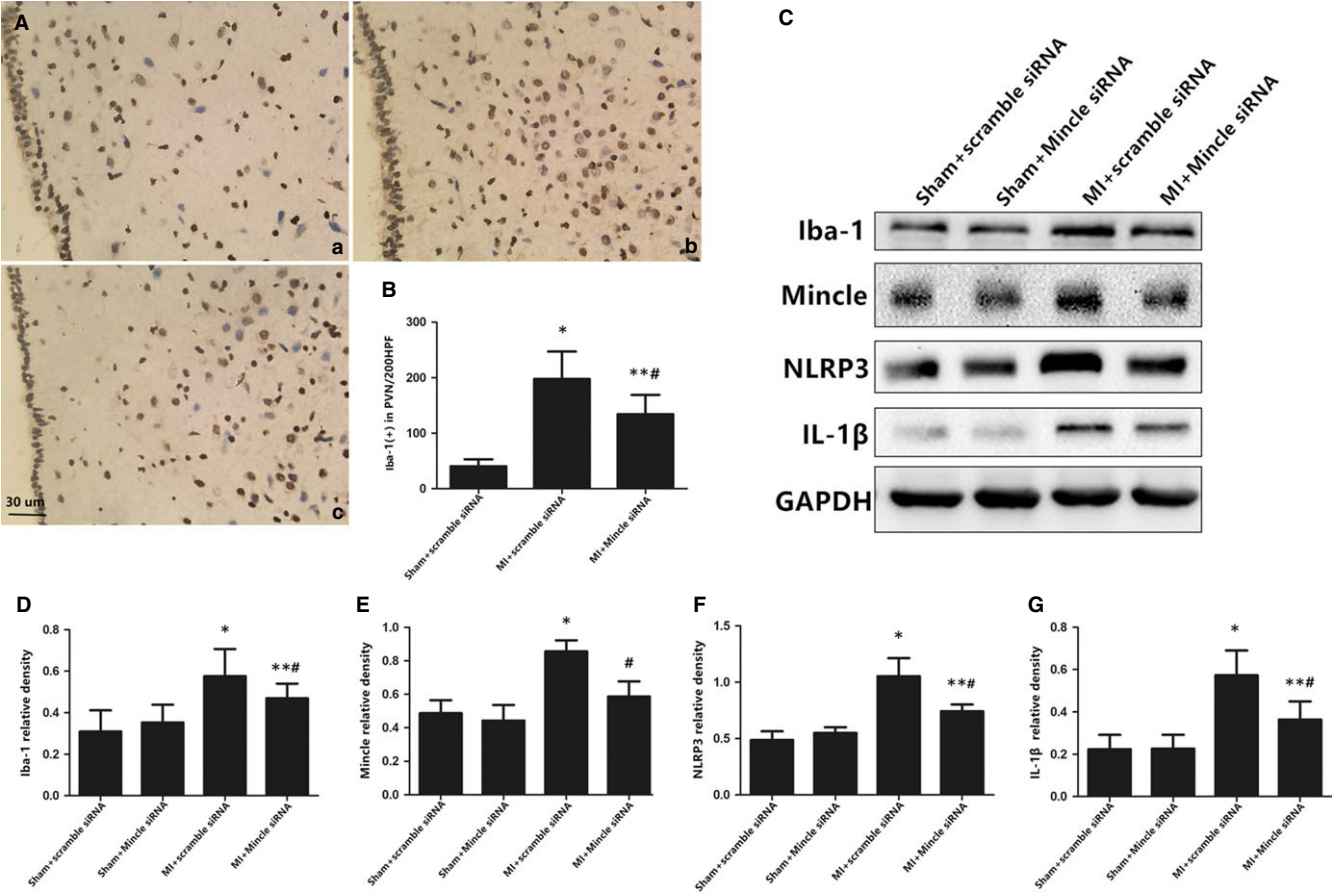
A, Representative immunohistochemical images of microglial activation at 24 h post‐MI, as indicated by the activation marker Iba‐1 (brown), and nuclei (blue) were stained with haematoxylin in the PVN (magnification ×200). a: the sham group; b: the MI + scramble siRNA group; c: the MI + Mincle siRNA group. C, Representative protein expression levels of Iba‐1 (17 kD), Mincle (25 kD), NLRP3 (120 kD) and IL‐1β (17 kD) as determined by western blot. Protein levels were quantified relative to GAPDH (37 kDa) levels (D‐G). The data are expressed as the mean ± SD. Bar = 30 μm. n = 10‐11 for each experiment and each group. *P < 0.01 and **P < 0.05 vs sham groups; ^#^ *P* < 0.05 vs MI+ scramble siRNA group

To reveal the role of Mincle within the PVN in the pathogenesis of MI, siRNA–based Mincle‐knockdown was performed as described in protocol 2. We enrolled 176 rats (eleven rats died) in the study. Iba‐1 protein was restrictedly located in microglia both in vitro and in vivo,^30^ therefore, we measured Iba‐1‐positive staining via immunohistological chemistry to evaluate the degree of the microglial activation post‐MI. Iba‐1 expression was increased significantly at 24 hours after MI, specifically when compared with the sham groups, and the PVN fraction exhibited the highest microglial expression by far, suggesting that microglia were located in the PVN area following MI (Figure 2A). Infarction induced marked Iba‐1 expression within the PVN and was slightly attenuated by Mincle siRNA as evaluated by western blot. We can conclude that Mincle silence may play a role for the activation of microglia during MI. A similar tendency was observed in the expression of Mincle receptor, NLRP3 and IL‐1β protein levels at 24 hours post‐MI. The administration of Mincle siRNA efficiently blunted the high levels of these proteins in the MI rats (Figure 2C‐G).

### 3.3 Mincle was distributed in microglia within the PVN after MI induction

**Figure 3:**
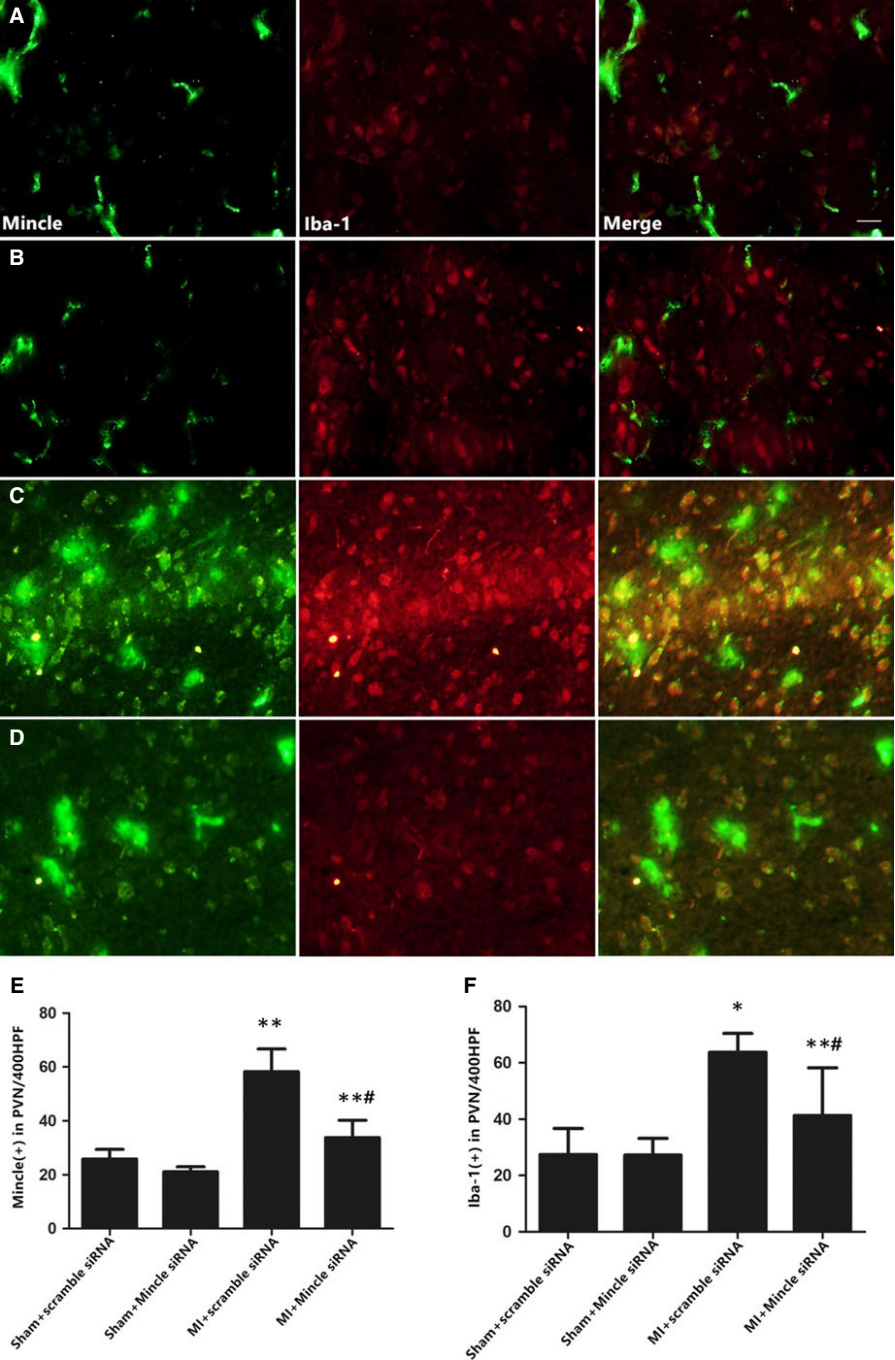
Representative double‐immunostaining for Mincle (green) and Iba‐1 (red) showed a limited distribution of Mincle on microglia within the PVN in the sham+ scramble siRNA (A), sham+ Mincle siRNA (B), MI+ scramble siRNA (C), MI+ Mincle siRNA (D) groups (magnification ×400). Bar = 30 μm. Quantity of Mincle‐positive (E) and Iba‐1‐positive (F) microglia were calculated. n = 10‐11 in each group. Each column with a bar represents the mean ± SD. *P < 0.01 and **P < 0.05 vs sham groups; ^#^ *P* < 0.05 *versus* MI+ scramble siRNA group

Double‐staining immunofluoresence was conducted in our study. Mincle was seen as green fluorescence, and Iba‐1 was seen as red fluorescence. We observed a similar tendency in Mincle and Iba‐1 expression via immunofluorescence. The presence of Mincle receptor was almost co‐localized with microglia within the PVN (Figure 3).

### 3.4 Mincle was involved in sympathetic hyperactivity in MI rats

**Figure 4:**
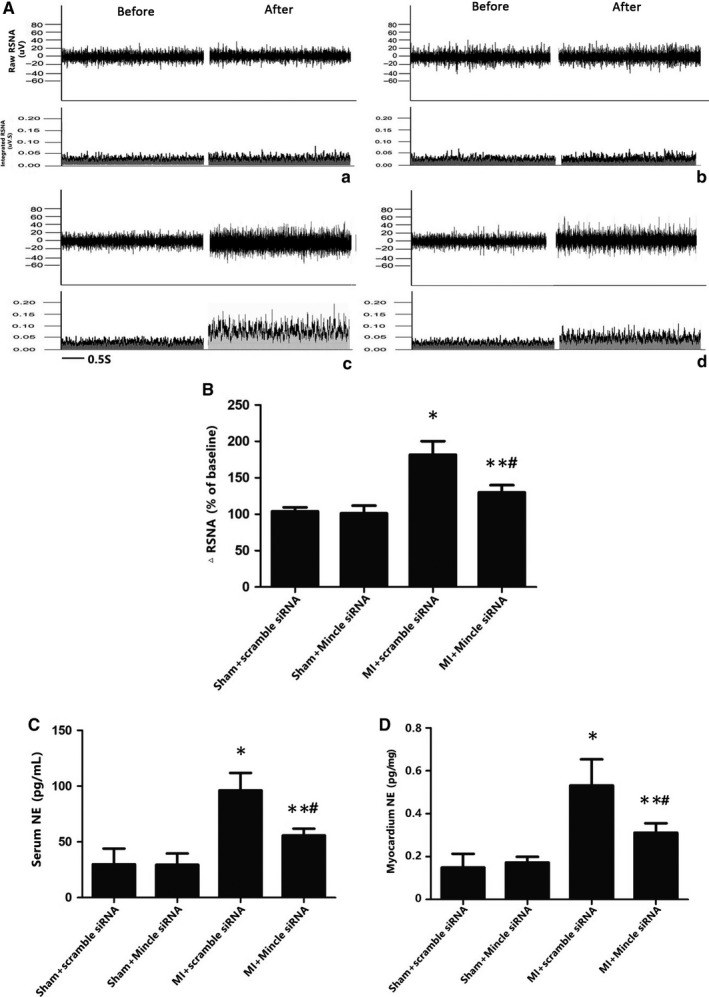
(A) Direct recordings of raw RSNA and integrated RSNA from the rats in the sham+ scramble siRNA (a), sham+ Mincle siRNA (b), MI+ scramble siRNA (c), MI+ Mincle siRNA (d) groups. The cytokine levels of NE from the serum (C) and myocardium tissue (D) as measured by ELISA. The results are shown as the mean ± SD. in the two independent experiments. n = 7‐9 in each group. *P < 0.01 and **P < 0.05 vs sham groups; ^#^ *P* < 0.05 vs MI+ scramble siRNA group

**Figure 5:**
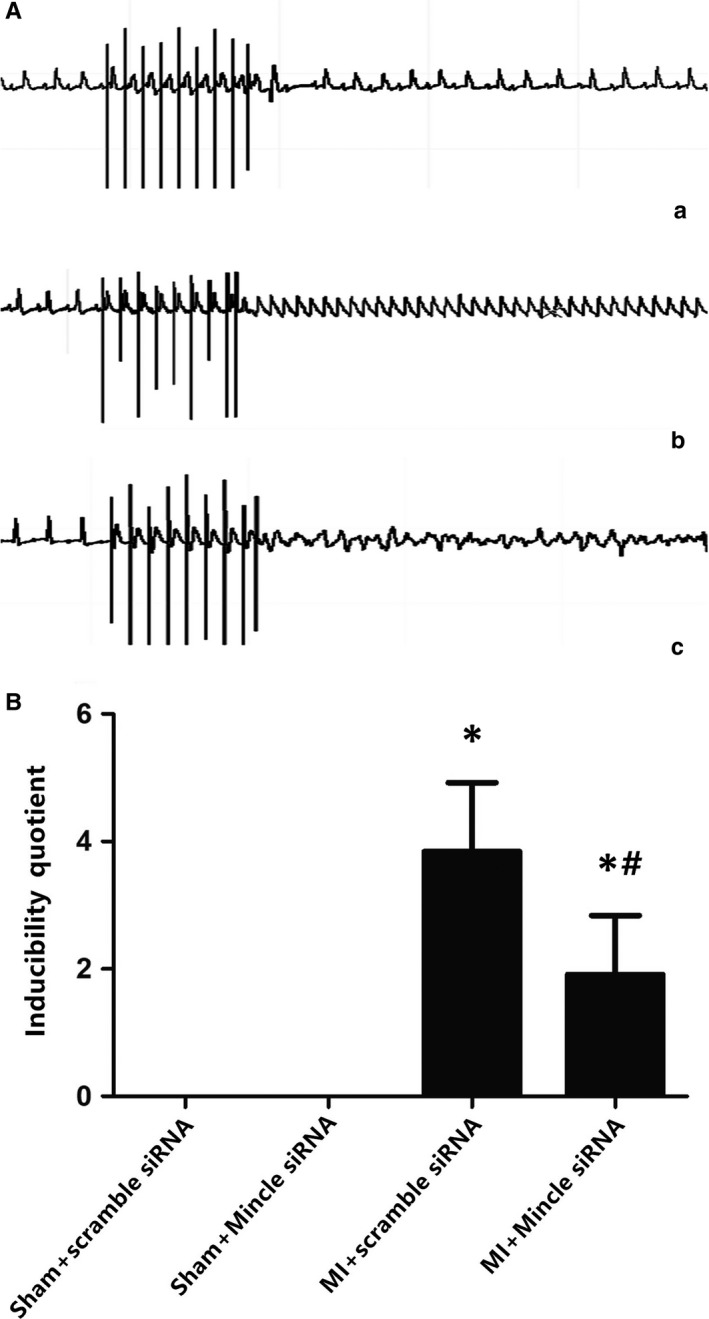
A, Programmed electrical stimulation at 24 h post‐MI. Ventricular premature beat (a), ventricular tachycardia (b) and ventricular fibrillation (c). B, Recordings of typical inducible VAs. Comparisons of the arrhythmia scores among four groups. n = 7‐8 in each group. *P < 0.01 compared with sham groups; ^#^ *P* < 0.05 compared with MI+ scramble siRNA group

The data depicted above supported the hypothesis that inhibition of Mincle receptor could be sufficient to blunt microglial expression as well as NLRP3 and IL‐1β activation post‐MI. Then, we investigated the sympathetic activity and biological function via analysing the levels of NE, a neural transmitter secreted by the sympathetic nerve, in the peripheral blood and myocardium as well as by measuring the RSNA, which indicates the activity of the efferent sympathetic nerve, heart rate variability (HRV) and programming electrical stimulation. We observed that MI could induce an increase in NE in both the plasma and the myocardium. NE secretion in the Mincle‐knockdown treated rats was markedly lower compared with that in the MI models (Figure 4C,D). The NE levels mirrored the RSNA content (Figure 4B). A higher VA score was also observed via electrical stimulation, suggesting sympathetic nerve hyperactivity (Figure 5). The HR, which clinically indicates sympathetic nerve excitation,^31^ was notably increased in the MI rats vs the baseline in the sham rats. PVN microinjection of Mincle siRNA in the AMI‐treated rats decreased the HR (Table 1).

**Table 1:** Protocol 2: HRV measurements at the end of study

Parameters	Sham		MI	
	Scramble siRNA	Mincle siRNA	Scramble siRNA	Mincle siRNA
No. of surviving rats	38	41	46	51
HR, bpm	395 ± 10.2	409 ± 14	443 ± 18.5^a^	422 ± 13.2^a^, ^b^
LF, nu	22.52 ± 3.18	20.64 ± 4.01	41.24 ± 6.06^a^	33.91 ± 3.58^a^, ^b^
HF, nu	88.21 ± 3.52	83.96 ± 4.38	70.00 ± 5.21^a^	77.89 ± 5.09^a^
LF/HF	0.2462 ± 0.05	0.2232 ± 0.03	0.5774 ± 0.03^a^	0.4179 ± 0.04^a^, ^b^

HR, heart rate; LF, low frequency; HF, high frequency; LF/HF, low frequency/high frequency.

Values are mean ± SD.

a *P < *0.05 compared with sham groups.

b *P < *0.05 compared with group MI+scramble siRNA.

### 3.5 Mincle induces sympathetic hyperactivity via the NLRP3/IL‐1β dependent pathway

**Figure 6:**
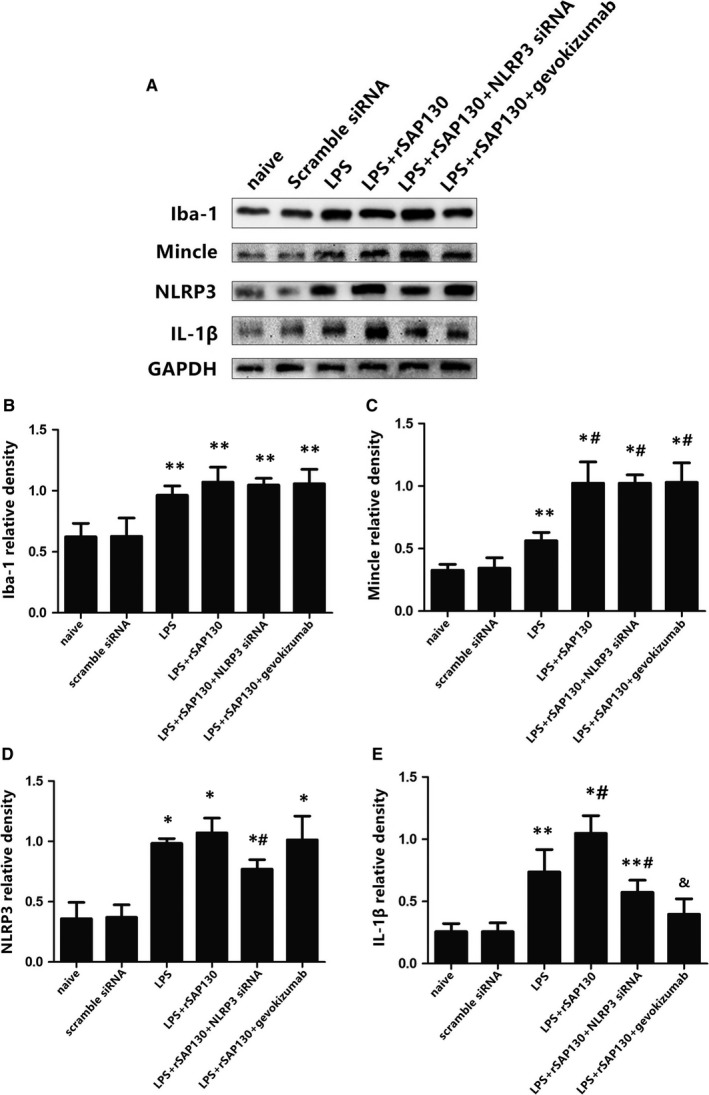
(A) Representative protein expression levels of Iba‐1 (17 kD), Mincle (25 kD), NLRP3 (120 kD), and IL‐1β (17 kD) as determined by western blot. Protein levels were quantified relative to GAPDH (37 kD) levels (B‐E). n = 14~16 in each group.*P < 0.01 and **P < 0.05 vs naïve group; ^#^ *P* < 0.05 vs LPS group; ^&^ *P* < 0.01 vs LPS+rSAP130 group

In protocol 3, we used several rats without any experiments as a real control group to compare with scramble siRNA‐microinjection‐treated groups. There were no significant differences observed between naïve group and scramble siRNA group, therefore we could exclude the potential effects of microinjection itself. It is reported that recombinant SAP130 can cooperate with LPS as a synergistic effect to induce robuster inflammatory gene expression and inflammasome activation in macrophages.^32^ Therefore, recombinant SAP130 was applied locally with LPS to stimulate Mincle in the PVN. We observed that Mincle receptor and IL‐1β protein expression were significantly increased by LPS+rSAP130 stimulation compared with LPS group, while there was no statistically difference in Iba‐1 and NLRP3 levels between LPS+rSAP130 and LPS stimulated rats. We hypothesized that LPS has already been a potent stimulator for microglial activation but not for Mincle receptor. In contrast, the inhibition of NLRP3 or IL‐1β did not affect the expression of Mincle and microglia in the LPS+rSAP130‐treated rats (Figure 6).

**Figure 7:**
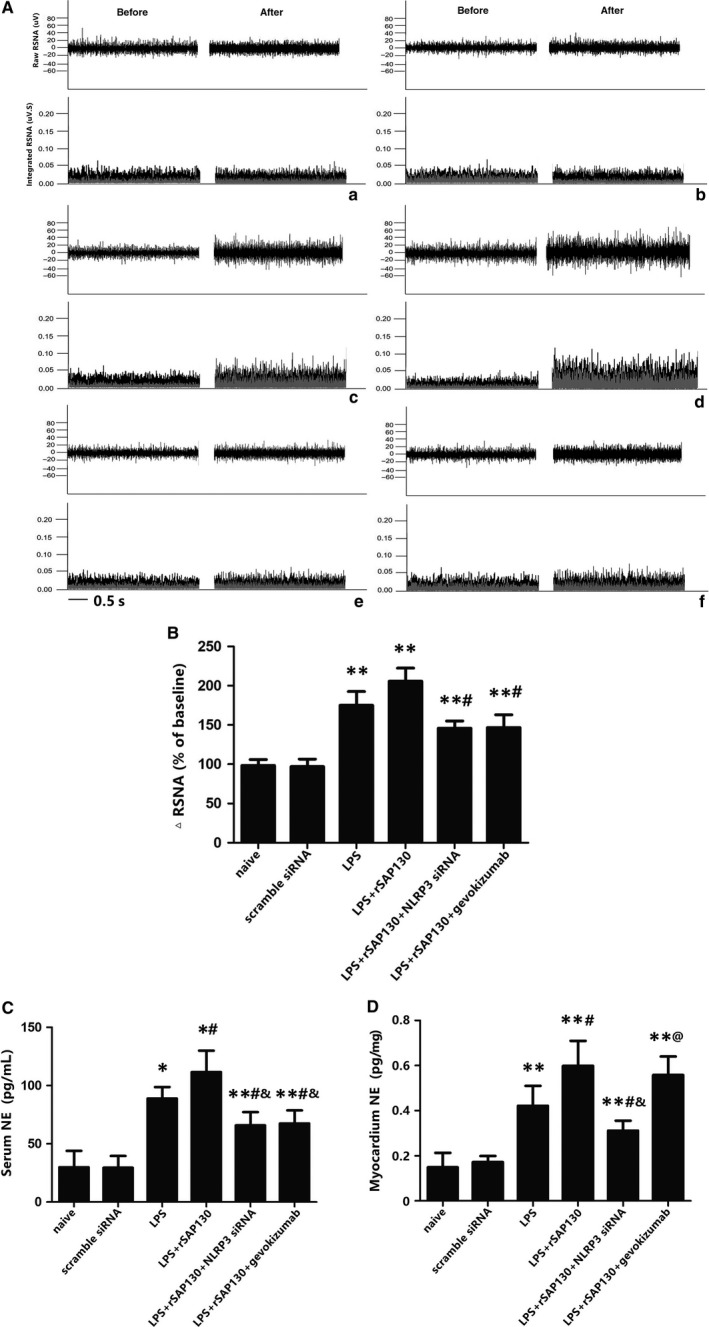
(A) Direct recordings of raw RSNA and integrated RSNA from rats in the naïve group (a), scramble siRNA group (b), LPS group (c), LPS+rSAP130 (d), LPS+rSAP130 + NLRP3 siRNA (e) and LPS+SAP130 + gevokizumab group (f). The cytokine levels of NE from serum (C) and myocardium tissue (D) as measured by ELISA. The data are expressed as the mean ± SD. of two experiments. n = 14‐16 in each group. *P < 0.01 and **P < 0.05 compared with the naïve group; #*P* < 0.05 compared with the LPS group; ^&^ *P* < 0.05 vs LPS+rSAP130 group; ^@^ *P* < 0.05 vs LPS+rSAP130 + NLRP3 siRNA group

Next, we explored the mechanism of peripheral sympathetic hyperactivity via Mincle activation in the PVN. Our data showed that LPS+rSAP130 significantly increased the NE levels in the plasma and myocardial tissue (Figure 7C,D) and the RSNA (*P* < 0.05, Figure 7B); this tendency was partly attenuated by the administration of NLRP3 siRNA or IL‐1β antagonist in serum NE concentration and the RSNA level. We could speculate that Mincle in the PVN participated in sympathetic nerve activity at least partly via the NLRP3/IL‐1β pathway.

## 4 DISCUSSION

This study reported the novel findings that MI induces the activation of Mincle expressed in microglia within the PVN, further causing sympathetic hyperactivity, and this process is partly associated with the NLRP3/IL‐1β pathway.

Rapid life‐threatening VAs including ventricular tachyarrhythmia(VT) and/or ventricular fibrillation(VF) lead to increased mortality and lethality following AMI.^33^ In the recent study, arrhythmic mechanism is different compared with previous studies which were investigated in the chronic stage of MI. Ventricular arrhythmias result from cardiac sympathetic hyperinnervation, induced by inflammation‐associated NGF elevation.^25^ While in the early stage of MI (before 7 days), increased sympathetic nerve activity responding to decreased myocardial oxygen supply, damaged myocardium and anxiety is a major cause of this condition. Previous studies demonstrated that there is a 100‐fold increase in extracellular catecholamines within the ischaemic zone 15 minutes after ischaemia. Catecholamine supersensitivity is responsible for electromechanical instability and ventricular arrhythmia.^34^ A growing body of evidence showed that inflammation occurring in PVN is related with multiple cardiovascular diseases including MI, while the central mechanism between inflammation in PVN and sympathetic hyperactivity post‐MI needs further investigation.

Mincle is a key C‐type lectin receptor and was originally found based on its strong induction in macrophages by inflammatory stimuli.^35^ It is rarely expressed in the normal circumstances, however, it can be activated powerfully following recognizing various stimuli, including microbial products or endogenous signals of tissue injury, such as debris from apoptotic and necrotic cells, heat shock proteins and nucleic acid fragments, then lead to macrophage excitation and induce a local inflammatory response.^36^ Previous studies demonstrated that Mincle plays an essential role in the pathologic process of acute renal inflammation, alcohol‐induced liver injury, traumatic brain injury and SAH,^9^, ^10^, ^12^, ^37^ however, little is known underlying the role of Mincle in sympathetic regulation in MI rats. To determine the effects of activated Mincle on sympathetic nerve activity post‐MI, Mincle siRNA was administered. It is reported that various “RNA interference (RNAi) therapeutics” have been applied in clinic trials.^38^ The superiority in a Mincle‐specific siRNA is to ensure the protective effects for experimental animals and eliminative results for a longer term inhibition in vivo. We observed an increase in RSNA, which is representative of enhanced sympathetic activity, and increased HRV, which may indicate dysfunction of the cardiac autonomic nervous system, following MI. MI‐induced VAs are hypothesized to be related to cardiac sympathetic overdrive; we analysed the NE content in the myocardium to test this hypothesis. Knockdown Mincle could induce a reduction in NE in the myocardium, and the ventricular arrhythmia score was reduced, supporting the concept that the VF threshold could be reduced when confronted with sympathetic blockade or vagal up‐regulation in animal studies.^39^ We further explored the mechanism of the PVN Mincle in sympathetic activation.

SAP130, an endogenous ligand for Mincle, is normally related with assembling the spliceosome and consisting of the U2 small nuclear ribonucleoprotein‐associated protein complex.^40^ Under steady condition, neither SAP130 nor Mincle is expressed at a high level.^41^ We noted that there was an increase in SAP130 release within the PVN post‐MI, however, the source of that need more exploration. SAP130 released from injured cells post‐MI may be one of the mechanism. Another possibility is that SAP130 is secreted through vesicles from macrophages.^42^ One previous study reported that rSAP130 administration markedly increased the expression of IL‐1β, however, there was no changes in Mincle expression after SAH.^12^ It suggested that Mincle might have various self‐ and non–self‐ligands other than SAP130. SAP130 has been identified to activate Mincle signalling in macrophages in vitro,^12^ however, no abundant evidence was found that exogenous SAP130 alone may active Mincle in vivo of naïve rats, therefore we used LPS and recombinant SAP130 to investigate the mechanism of Mincle function in vivo. We observed that Mincle was activated in parallel with NLRP3 expression increase as well as IL‐1β release after LPS+rSAP130 administration, and the RSNA was high as well as NE increased while they were both inhibited by NLRP3 siRNA or IL‐1β inhibitor. IL‐1β plays an essential role in regulating various physiological, behavioural, and endocrine actions in the CNS.^43^ It is reported that central microinjection of IL‐1β could promote the release of serum NE and increase blood pressure.^44^ Different from most other pro‐inflammatory cytokines, however, IL‐1β is not synthesized via gene expression solely. The reason is that IL‐1β lacks a signal peptide and is not released through the default ER‐Golgi dependent mechanisms employed by other cytokines. It is synthesized and maintained as an inactive pro‐form in the cytoplasm. Intracellular signalling complexes called inflammasomes containing caspase‐1 are activated following sensing a distinct stimulus or set of stimuli.^45^, ^46^ Next, caspase‐1 cleaves pro‐IL‐1β into active and mature IL‐1β. Simultaneously caspase‐1 plays an important role in the secretion of IL‐1β, however, the mechanism remains poorly understood. Previous studies have reported several types of inflammasomes and the best‐characterized one is the NLRP3 inflammasome, which contains the sensor molecule NLRP3, the adaptor protein ASC, and pro‐caspase‐1.^46^, ^47^ Reports from us and others demonstrated that the NLRP3 inflammasome is related with several sterile inflammatory diseases, including obesity, atherosclerosis and MI.^25^, ^48^ On the other hand, Syk, the downstream protein of Mincle, regulates NLRP3 activity via the production of reactive oxygen species.^18^ Qi et al^23^ observed increased NLRP3 expression within the PVN in salt‐sensitive hypertension rats compared with the normal rats and chronic antagonism of IL‐1β in the PVN decreased the levels of NLRP3. However, we did not found that NLRP3 expression was decreased following administration of IL‐1β inhibitor. We hypothesized that there was no enough time for NLRP3 level changes in our experimental duration via IL‐1β inhibition.

Most pro‐inflammatory cytokines are too large to pass through the BBB; therefore, some studies have demonstrated that pro‐inflammatory cytokines, such as IL‐1β and TNF‐α, are produced mostly in the PVN by local microglia.^49^ Another study demonstrated that ischaemic heart tissue could release vascular adhesion molecules such as selectins and integrins, which play an important role in increasing the permeability of the BBB, allowing pro‐inflammatory cytokines produced by the heart tissue to come in contact with the brain tissue through the bloodstream.^50^ Makoto et al^35^ reported that the level of Mincle mRNA was significantly up‐regulated by several inflammatory stimuli including LPS,TNF‐α, and IL‐6 in wild‐type macrophages. We could speculate that except SAP130, cytokines such as TNF‐α and IL‐6 derived from ischaemic heart cross through the BBB to activate Mincle in microglia, resulting in pro‐inflammatory cytokines release and broader inflammatory response. A study found that the PVN has a denser vascularity than surrounding areas,^51^ and we verified this by immunohistochemical staining of CD34, a marker of vascular endothelial cells (supplemental data), providing a substrate for higher concentrations of stimuli like TNF‐α to activate more microglia. This is a possible interpretation of the distribution of activated microglia in the PVN. The reasons why pro‐inflammatory cytokine IL‐1β could affect sympathetic drive post‐MI are unknown. Previous studies have demonstrated that IL‐1β secretion promotes expression of activated nuclear transcription factor kappa B (NF‐κB), which can produce a variety of excitatory mediators including glutamate and NE in the PVN, which are involved in neuroendocrine regulation. IL‐1β also stimulates angiotensin II type 1 receptor and reactive oxygen species (ROS) production in many kinds of pathological conditions, then enhancing glutamatergic excitatory and attenuating GABAergic inhibitory activities from sympathetic neurons.^52^, ^53^ It was reported in heart failure and hypertensive rat models which may be also applied in MI rats.

### 4.1 Outlook

The present results indicate that Mincle expressed in microglia within the PVN is markedly activated in MI rats following sympatho‐excitation. The mechanism on the role of inflammation within the PVN in sympathetic regulation was previously conducted,^53^ however, our study constitutes a novel perspective exploring Mincle/NLRP3/IL‐1β pathway in the CNS and a specific experiment involving in the local blockage of Mincle and its downstream targets to demonstrate that inflammation within the PVN could participate in sympathetic hyperactivity post‐MI. The study provides a feasible solution to limiting the complications associated with MI. Mincle could communicate with another C‐type lectin receptor or toll like receptors by forming a heterodimer, which may expand ligand specificity and confer multiple functions.^54^ Therefore, our next study will primarily focus on the molecular pathway which induces Mincle expression and the connections between Mincle and other innate immune receptors in sympathetic hyperactivity after myocardial infarction.

### 4.2 Limitations

Firstly, a previous study showed that MI results in activation of microglia in the periaqueductal gray (PAG), rostral ventrolateral medulla (RVLM), nucleus tractus solitarius (NTS) and area postrema (AP) and PVN,^55^ indicating that the PVN is not the only cardiovascular regulatory nucleus for sympathetic activation. We did not determine whether microglia in the PVN induce sympathetic hyperactivity that is affected by other nerve nuclei. Secondly, stimulated astrocytes and neurons release pro‐inflammatory cytokines during various CNS diseases, such as Parkinson's disease and SAH.^12^, ^56^ However, little is known regarding whether astrocytes and neurons could express Mincle and release pro‐inflammatory cytokines such as IL‐1β post‐MI. Thirdly, administration of siRNA–based Mincle‐knockdown alleviates sympathetic nerve activity post‐MI, it is possible that the use of siRNA technique may also affect the microglial functional activities. Despite such limitations, our results point towards a critical lethal effect of Mincle receptor within the PVN in sympathetic hyperactivity following myocardial infarction.

## 5 CONCLUSIONS

Taken together, our results reveal that myocardial infarction elicits Mincle receptor activation expressed in microglia within the PVN, and that this activation induces inflammatory situation. Inhibition of Mincle ameliorates sympathetic hyperactivity that is meditated at least partly by NLRP3/IL‐1β pathway.

## CONFLICT OF INTEREST

None.
