# Supplementary material for: Microglial Mincle receptor in the PVN contributes to sympathetic hyperactivity in acute myocardial infarction rat
Source: J Cell Mol Med. 2018 Oct 24;23(1):112–25. doi: 10.1111/jcmm.13890 (PMC6307841; doi:10.1111/jcmm.13890)
Supplement: Supplementary file 1 [file JCMM-23-112-s001.docx]

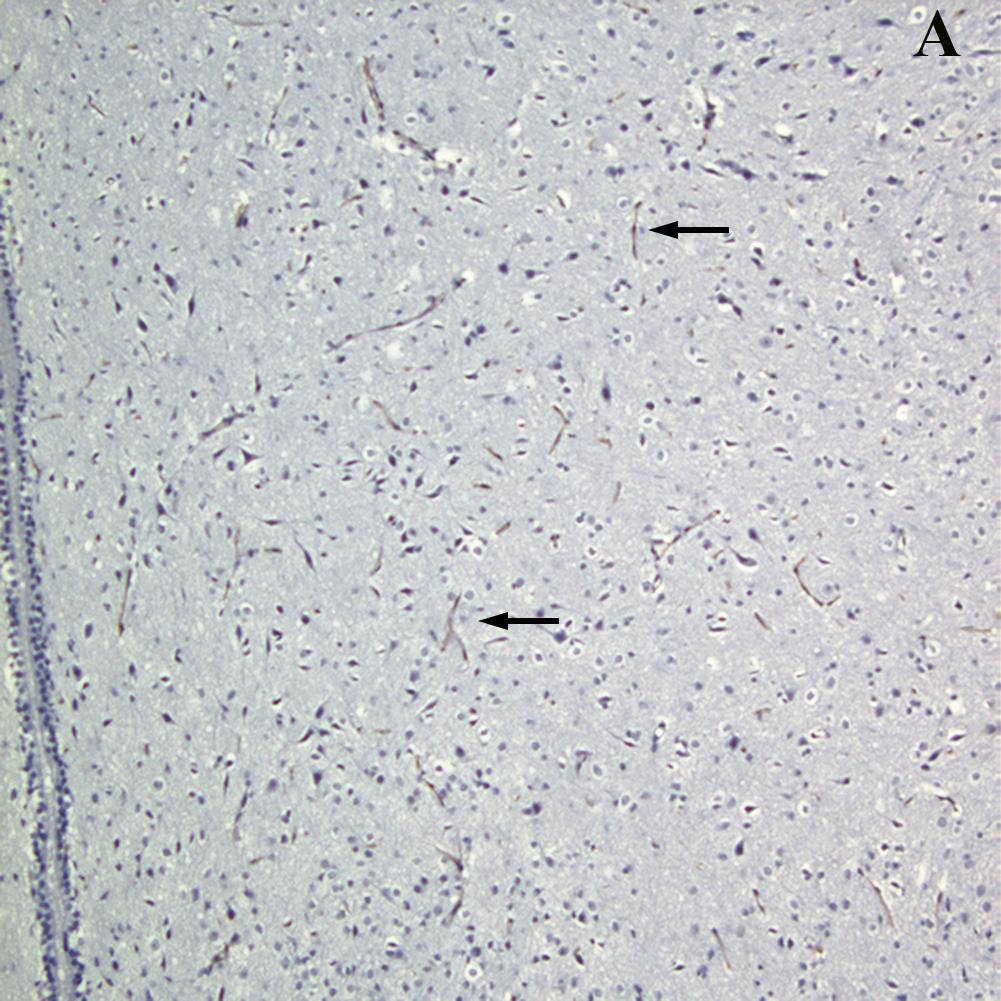


Fig.1 Immunochemistry staining of CD34 (a marker of vascular endothelial cells) in PVN in naive rats (magnification ×100). We showed there is dense vascularity of the PVN, consistent with previous study.


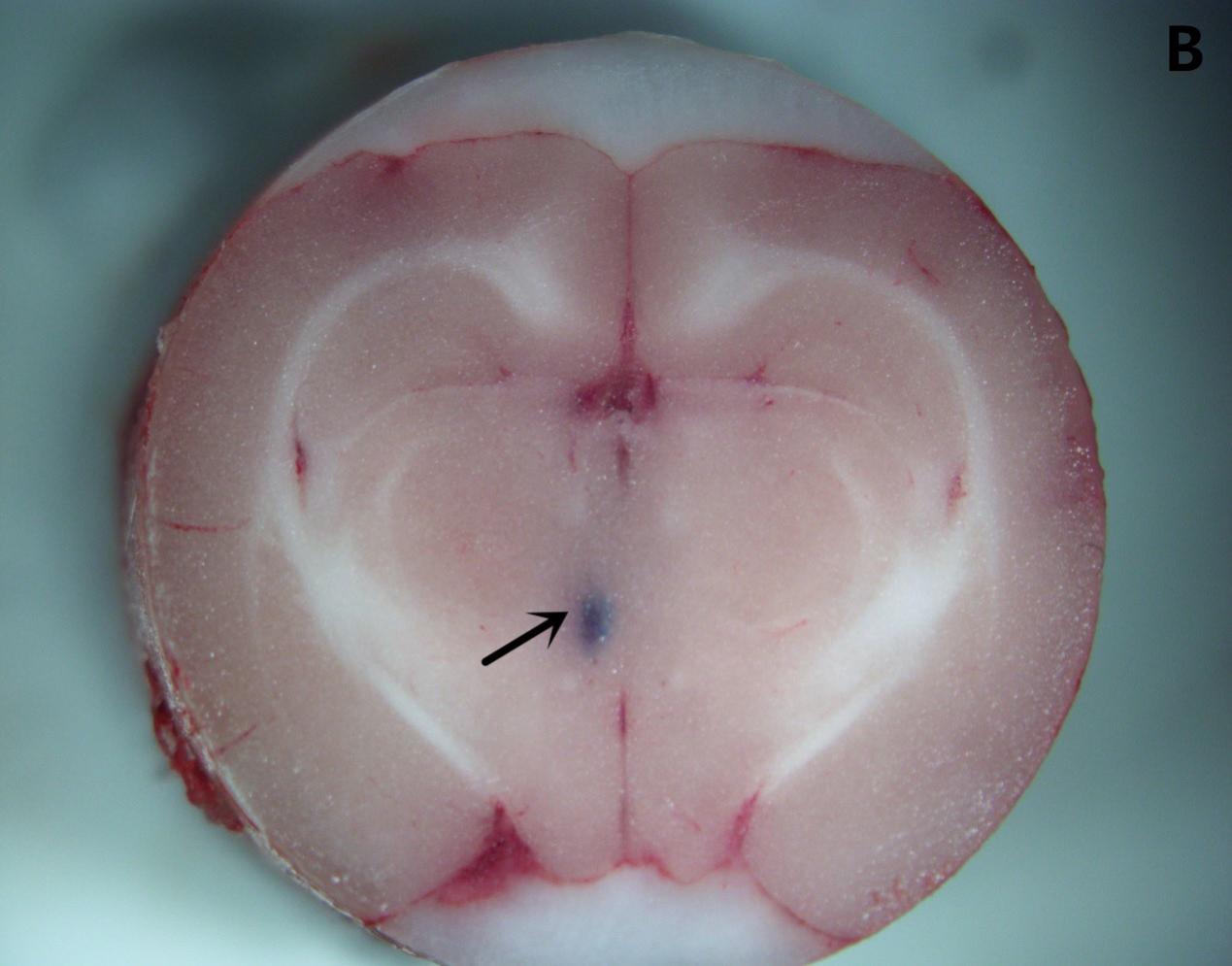


Fig.2 Original color image of PVN area was delineated after injection of methylene blue. The arrow pointed to PVN area, as indicated by the blue staining.


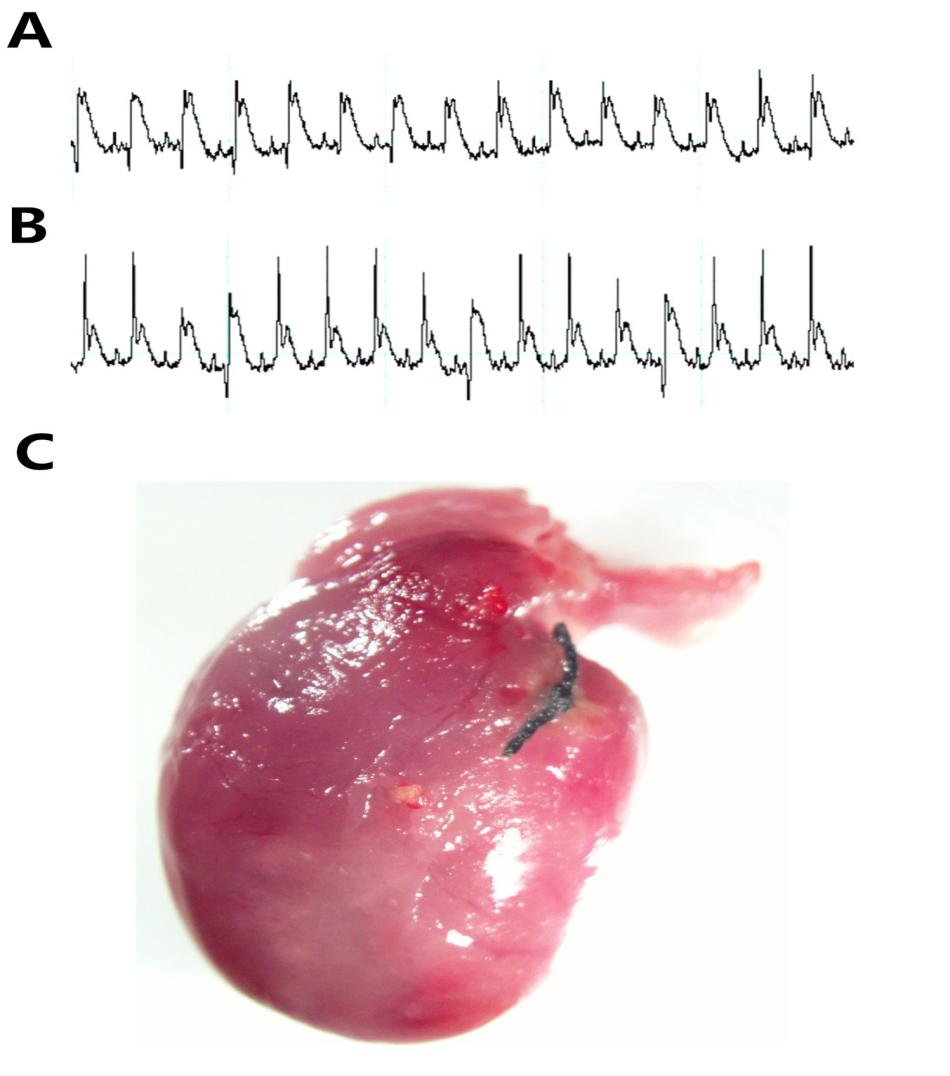


Fig.3 Confirmation of myocardial infarction by recorded ECG after MI surgery; elevated ST (A)，ventricular premature beat (B) and mottled and pale appearance of infarcted region by visualizing (C).


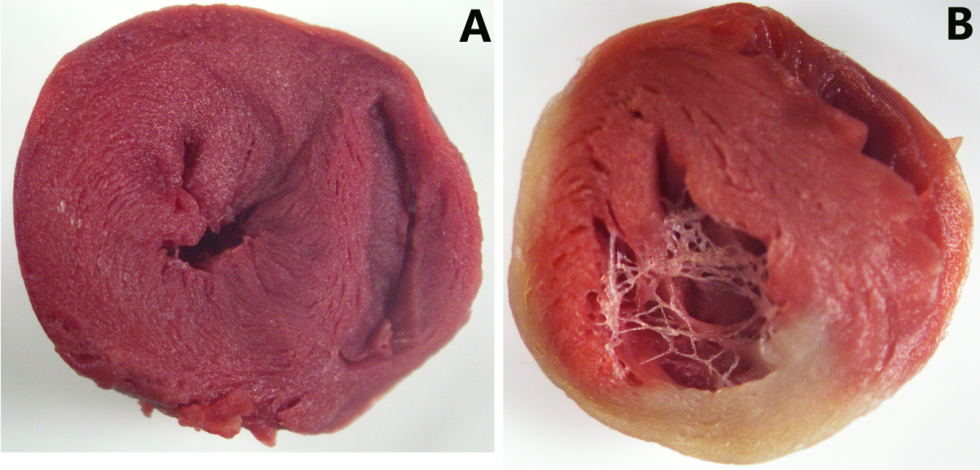


Fig.4 Representative images showing infarct areas in cross section slices, the infarct area was unstained while the undamaged area turned red: sham group (A) and MI group (B).


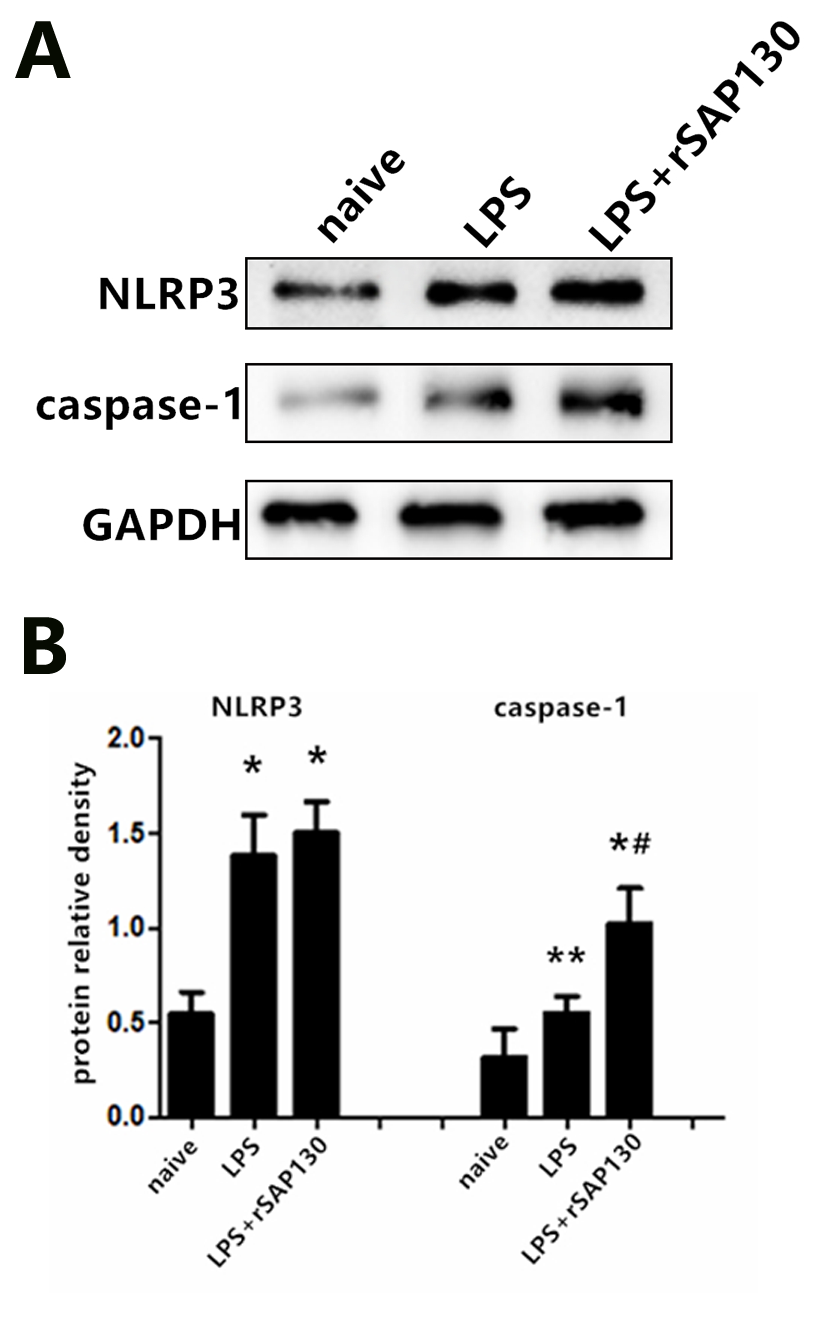


Fig.5 Representative protein expression levels of NLRP3 (120 kD) and Caspase-1 (17 kD) as determined by western blot (A). Protein levels were quantified relative to GAPDH (37 kD) levels (B). n=10 in each group. *P < 0.01 and **P < 0.05 *versus* naïve group; ^#^P < 0.05 *versus* LPS group.
